# Inhibition of acylglycerol kinase sensitizes DLBCL to venetoclax via upregulation of FOXO1-mediated BCL-2 expression

**DOI:** 10.7150/thno.72786

**Published:** 2022-07-18

**Authors:** Na Ning, Si Zhang, Qi Wu, Xun Li, Dong Kuang, Yaqi Duan, Minghui Xia, Huicheng Liu, Junmei Weng, Hongping Ba, Zhaohui Tang, Xiang Cheng, Heng Mei, Liu Huang, Qilin Ao, Guoping Wang, Yu Hu, Arian Laurence, Jing Wang, Guihua Wang, Xiang-Ping Yang

**Affiliations:** 1 Department of Immunology, School of Basic Medicine, Tongji Medical College, Huazhong University of Science and Technology (HUST), Wuhan, China; 2 Laboratory of Cardiovascular Immunology, Institute of Cardiology, Union Hospital, Tongji Medical College, HUST, Wuhan, China; 3 GI Cancer Research Institute, Tongji Hospital, Tongji Medical College, HUST, Wuhan, China; 4 Institute of Pathology, Tongji Hospital, Tongji Medical College, HUST, Wuhan, China; 5 Department of Pathology, School of Basic Medicine, Tongji Medical College, HUST, Wuhan, China; 6 Department of Surgery, Tongji Hospital, Tongji Medical College, HUST, Wuhan, China; 7 Institute of Hematology, Union Hospital, Tongji Medical College, HUST, Wuhan, China; 8 Department of Oncology, Tongji Hospital, Tongji Medical College, HUST, Wuhan, China; 9 Institute of Cellular Medicine, Newcastle University, Newcastle, United Kingdom

**Keywords:** DLBCL, Venetoclax, AGK, BCL-2, FOXO1

## Abstract

**Background:** Despite of the paradigm change on the treatments of acute myeloid leukemia (AML) and chronic lymphocytic leukemia (CLL) by venetoclax, it has been less successful in the treatment of diffuse large B-cell lymphoma (DLBCL). Here, we explored whether acylglycerol kinase regulates the sensitivity of DLBCLs to venetoclax and its mechanism in both cell lines and preclinical animal models.

**Methods:** The expression of AGK and sensitivity to venetoclax of seven DLBCL cell lines were determined. Upon knockdown and overexpression of AGK by lentivirus in DLBCL cells, the venetoclax-induced apoptosis and PTEN-FOXO1-BCL-2 signaling axis were evaluated *in vitro*. The efficacy of venetoclax and PTEN-FOXO1-BCL-2 signaling axis were evaluated in immunodeficient NCG mice that were implanted with control or shAGK stably transduced SU-DHL4 cells. The expressions of AGK, BCL-2 and FOXO1 were evaluated in tumor tissues of DLBCL patients.

**Results:** AGK expression was inversely correlated with sensitivity of DLBCL to venetoclax. Inhibition of AGK rendered the DLBCL cells more sensitive to venetoclax. Mechanistically, AGK phosphorylated and inactivated PTEN, which led to AKT activation and reduced FOXO1 nuclear translocation. Inhibition of AGK also led to enhanced efficacy of venetoclax for suppression of DLBCL tumor growth *in vivo*, which was dependent on FOXO1. In human DLBCL tumor tissues, the expression of AGK inversely correlated with BCL-2 expression, as well as the amounts of nuclear FOXO1.

**Conclusions:** Our data demonstrated that AGK regulates venetoclax response in DLBCL via PTEN-FOXO1-BCL-2 signaling axis. Targeting AGK may enhance the efficacy of venetoclax for the treatment of DLBCL patients.

## Introduction

Diffuse large B-cell lymphoma (DLBCL) is the most common type of non-Hodgins lymphoma (NHL) and represents a heterogeneous entity with different molecular characterizations and prognosis 1, 2. The standard care for DLBCL is rituximab plus cyclophosphamide, doxorubicin, vincristine, and prednisone (R-CHOP) 3. However, 40-45% patients are not responsive to the first-line therapy or relapse after an initial response 4.

B-cell leukemia/lymphoma-2 (BCL-2) is a key anti-apoptotic protein that regulates the intrinsic apoptosis pathway, which is often dysregulated in cancers 5, 6. One-third of patients with DLBCL harbor BCL-2 translocations and have enhanced BCL-2 expression, which is linked to poor outcome 7. Targeting BCL-2 family proteins has advanced clinical therapies for hematological malignancies 8, 9. Venetoclax (ABT-199) is a highly selective BCL-2 inhibitor 10, which was approved for patients with chronic lymphocytic leukemia (CLL)/small lymphocytic lymphoma (SLL) and in combination therapy with azacytidine or decitabine or low-dose cytarabine to treat adult acute myeloid leukemia (AML) patients, representing a paradigm change of AML treatment 11, 12.

Recently, a single-agent phase I trial of venetoclax in relapsed/refractory NHL reported an 18% overall response rate (ORR) in DLBCL patients 13. It is unclear why the ORR was low for DLBCL. Upregulation of MCL-1, BCL-XL or a reprograming of mitochondrial metabolism have all been suggested as causes 14-16. Enhancing the efficacy of venetoclax and identification of novel biomarkers to predict venetoclax responses are unmet needs for the treatment of DLBCL.

Recently, it has been reported that reprogramming mitochondrial metabolism is linked to venetoclax resistance and the signal pathways related to mitochondrial structure and function and lipid metabolism were significantly enhanced in Venetoclax-resistant cell lines 16. Initially identified as a mitochondrial lipid kinase, acylglycerol kinase (AGK) catalyzes the phosphorylation of monoacylglycerol and diacylglycerol to lysophosphatidic acid and phosphatidic acid, respectively 17. Mutations of *AGK* gene cause Sengers syndrome, an autosomal recessive disorder with congenital cataracts, hypertrophic cardiomyopathy, skeletal myopathy and lactic acidosis, through its role in mitochondrial function and metabolism 18, 19. AGK regulates multiple intracellular signaling pathways and is highly expressed in numerous types of tumors, including prostate cancer, breast cancer, cervical squamous cell carcinoma, and esophageal squamous cell carcinoma 20. Recently, it has been shown that AGK potentiates JAK2-mediated STAT3 activation and cell proliferation of both megakaryocytes and nasopharyngeal carcinoma cells independent of its kinase activity 21, 22. In addition, AGK phosphorylates PTEN and promotes PI3K-AKT-mediated glycolysis and anti-tumoral activities of CD8^+^ T cells 23.

Here, we identified that AGK was enriched in signaling pathways of mitochondrial function and lipid metabolism that were involved in venetoclax resistance. Suppression of AGK expression sensitized DLBCL cells to venetoclax-induced apoptosis both *in vitro* and *in vivo*. Mechanistically, AGK phosphorylated and inactivated PTEN, leading to enhanced PI3K-AKT activation, reduced FOXO1 nuclear localization and its target gene *BCL-2* expression. Inhibition of AGK expression in DLBCL cells sensitized them to venetoclax in a FOXO1-dependent manner. Furthermore, BTK inhibitor, ibrutinib, mediated sensitization to venetoclax through AGK pathway. Together, our data identify the combination of AGK suppression may enhance the efficacy of venetoclax for the treatment of aggressive DLBCL.

## Materials and methods

### Human tissue samples

33 DLBCL patients were histopathological diagnosed and lacked prior therapy. Upon surgical removal, tumor tissues were stained for BCL-2 and divided into BCL-2^high^ (4 GCB and 12 ABC) and BCL-2^low^ (10 GCB and 7 ABC) groups by two independent pathologists. High expression of BCL-2 was defined as expression by ≥ 5% of malignant cells. All patients provided written informed consent approving the use of their samples under Institutional Review Board approval.

### Mice

NOD/ShiLtJGpt-*Prkdc^em26^Il2rg^em26^/*Gpt (NCG) mice were purchased from Gempharmatech (Nanjing, China) and maintained at a specific-pathogen free facility under 12 h light-dark cycle. All procedures were conducted in accordance with the Institutional Animal Care and Use Committee of Tongji Medical College.

### Cell culture

Human DLBCL cell lines SU-DHL2 from DSMZ, OCI-LY1, TMD8, SU-DHL4, SU-DHL6, SU-DHL10, and SU-DHL16 from ATCC were cultured in RPMI 1640 supplemented with 10% FBS. HEK293T cells were cultured in DMEM medium with 10% FBS. All cells were cultured at 37 °C in a 5% CO_2_ atmosphere.

### RNA-sequencing analysis

Microarray profiling data of lymphoma cell lines were obtained from the Gene Expression Omnibus (GEO) database under accession number GSE128563. Two replicates of venetoclax resistant cell line (GSM3680166-3680167) and parental cell line (GSM3680164-3680165) based on the AffymetrixGPL11154 platform were analyzed. R package clusterProfiler was used to identify enriched pathways in gene ontology (GO) analysis. Differentially expressed genes with a cutoff of log_2_FC > 1 were selected to identify enriched biological processes. Enriched pathways were selected by a cutoff of *p* value < 0.05.

### Cell viability assay

3×10^4^ DLBCL cells were plated onto 96-well plates and incubated with varying amounts of venetoclax (MedChem Express, HY15531), or ibrutinib (MedChem Express, HY10997) for 72 h at 37 °C. Cells were incubated with 20 μL Cell Counting Kit-8 (CCK-8) solution (biosharp, BS350B) for 3 h before measuring the absorbance at 450 nm. The cell viability was calculated by the formula: Cell viability (%) = [OD (drug^+^) - OD (Blank)] / [OD (drug^-^) - OD (Blank)] × 100%. The IC50 concentrations of inhibitors were determined using GraphPad Prism 7 (La Jolla, CA) by log(inhibitor) vs. normalized responses.

### Lentivirus production and viral transduction

For shRNA-mediated AGK knockdown, scramble shRNA and AGK shRNA were cloned into pLKO.1 vector. Sequences for scrambled and AGK shRNA were as follows: shRNA AGK#1 (TRCN0000153242): 5'-CCGGGCCCTTCCATTTCTCTTCTTTCTCGAGAAAGAAGAGAAATGGAAGGGCTTTTTTG; shRNA AGK#2 (TRCN0000153828): 5'-CCGGCCTCAACTGTACTTGGAGAAACTCGAGTTTCTCCAAGTACAGTTGAGGTTTTTTG; Full-length cDNA encoding AGK was cloned into Murine Stem Cell Virus-IRES-Thy1.1 (MSCV-IRES-Thy1.1, RV) vector (Addgene, #17442) using *Nhel* and *Ecor1* enzymes. For lentivirus production, lenti-viral shRNA constructs, pMD2.G (Addgene, #12259) and psPAX2 (Addgene, #12260) were transfected at a 3:1:2 ratio into HEK293T cells of 80-90% confluency using Lipofectinmin 2000. Supernatants containing viral particles were collected and filtered at day 2-3 after transfection. Cells were incubated with lentiviral particles in the presence of 10 μg/mL polybrene, followed by selection with 5 μg/mL puromycin for 14 days.

### Immunoblotting

Cells and tumor tissues were lysed with RIPA lysis buffer. Equal amounts of proteins were separated by SDS-PAGE electrophoresis and then transferred onto PVDF membranes (Millipore). After blocking with 5% milk for 2 h, the blots were probed with the anti-AGK (Abcam, ab137616), anti-BCL-2 (Abclonal, A19693), anti-PTEN (CST, #9188), anti-pPTEN (CST, #9551), anti-AKT (CST, #4691), anti-pAKT(T308) (CST, #2965), anti-pAKT (S473) (CST, #4060), anti-FOXO1 (CST, #2880), anti-pFOXO1 (CST, #9464), anti-β-Actin (CST, #4970) and anti-GAPDH (Servicebio, GB11002) antibodies overnight at 4 °C. Then, the membrane was incubated with a horseradish peroxidase-conjugated secondary antibody for 1.5 h, followed by ECL detection (GE Healthcare).

### Analysis of apoptosis by flow cytometry

The percentages of apoptotic cells were determined 48 h after venetoclax treatment. 1×10^6^ cells of shRNA control and shRNA AGK#1 groups were stained with propidium iodide (Sigma, P4170) and FITC conjugated Annexin V (Biolegend, 640906) for 15 min. Samples were analyzed on a BD FACSVerse flow cytometer and data were analyzed using FlowJo software.

### Cell fractionation

The cytosolic and nuclear fractions of SU-DHL4 cells were isolated using nuclear and cytoplasmic protein extraction kit (Beyotime, P0027). In brief, 2×10^6^ cells were incubated with 200 μL reagents A on ice for 15 min, followed by addition of 10 μL reagents B and incubation on ice for another 1 min. Lysis were centrifuged at 12,000 rpm for 5 min and supernatants were collected as cytoplasmic fractions. The cell pellets were resuspended with 30-50 μL nucleoprotein extraction reagent, followed with vigorous shaking for 15-30 s every 2 min in total of 30 min. Nuclear fractions were obtained upon centrifugation at 12000 rpm for 10 min.

### Chromatin-immunoprecipitation assay

Chromatin immunoprecipitation (CHIP) was performed with CHIP assay kit (Beyotime, P2078) according to the manufacturer's description. In brief, SU-DHL4 cells were harvested followed by cross-linking with 1% (v/v) formaldehyde for 10 min and resuspended in SDS lysis buffer. Cells were lysed and DNAs were fragmented by sonication. The chromatins were immunoprecipitated with anti-FOXO1 overnight at 4 °C. After washing and elution, crosslinks were reversed for 4 h at 65 °C. The eluted DNA was purified and analyzed by qPCR using a Bio-Rad SYBR Green intercalating fluorophore system with *BCL-2* primers forward: GTGTAGTGCGCGGACACCTAGG and primers reverse: GCTGCCCTGCTGTGAAGACAGG.

### *In vivo tumor xenograft* model

Human shRNA control and shRNA AGK#1 SU-DHL4 cells (2×10^7^/100 μL PBS) were mixed with 1:1 ratio of Matrigel™ and implanted subcutaneously into the flank of six weeks old female NCG mice. Once tumors reached approximately 60-100 mm³, mice were gavaged with 100 mg/kg of ABT199 daily for 8 days that was formulated as a suspension in PEG300 and saline (1:1 v/v). All mice were sacrificed at day 12 and tumors were collected. For FOXO1 inhibition, AS1842856 was dissolved in the saline and HP-β-CD (15% w/v), mice were intragastrically administered with 100 mg/kg twice a day, every two days for a total of 10 days. Tumor volumes were calculated from bilateral caliper measurements and calculated according to the formula “tumor volume = length × width^2^/2”.

### Immunohistochemistry and immunofluorescence

Mouse tumor tissues were isolated, formalin fixed, paraffin embedded and cut into serial histologic sections, and stained with hematoxylin and eosin. Immunohistochemistry was performed with antibodies against cleaved-caspase3 (Wanlei, WL02117), FOXO1 (CST, #2880), Ki67 (Abcam, ab1667), α-SMA (Bioss, BS-10196R) and BCL-2 (Abclonal, A19693). TUNEL staining was performed with TUNEL label solution (Roche, G1501). DAPI was used for nuclear counterstaining for 10 min. Tissue sections from DLBCL patients were stained with BCL-2 (Abclonal, A19693), AGK (Invitrogen, PA5-28566) and FOXO1 (CST, #2880). Percentages of positive areas of different sections were determined by Image J.

### Statistics analysis

Data were graphed using GraphPad Prism software. Statistical significance was determined by unpaired Student's *t* test, one-way ANOVA or two-way ANOVA. Correlation was assessed using Spearman correlation analysis. (*P < 0.05; **P < 0.01; ***P < 0.001; ns, not significant).

## Results

### The expression of AGK was inversely correlated with sensitivity of DLBCL to venetoclax

To identify potential regulator of venetoclax sensitivity, we analyzed the transcriptome expression of two parental cell lines and two venetoclax-resistant cell lines and identified enriched pathways using gene ontology (GO) analysis. We confirmed that signal pathways related to mitochondrial structure and function and lipid metabolism were significantly enhanced in venetoclax-resistant cell lines (Figure S1A). By cross examination of the molecules involved in the related pathways, we found *PRKAA*,* SIRT4, AKT1,* and *AGK* were shared by multiple mitochondrial signaling pathways, among which *AGK* was involved in all four related signaling pathways (Figure S1B).

**Figure 1:**
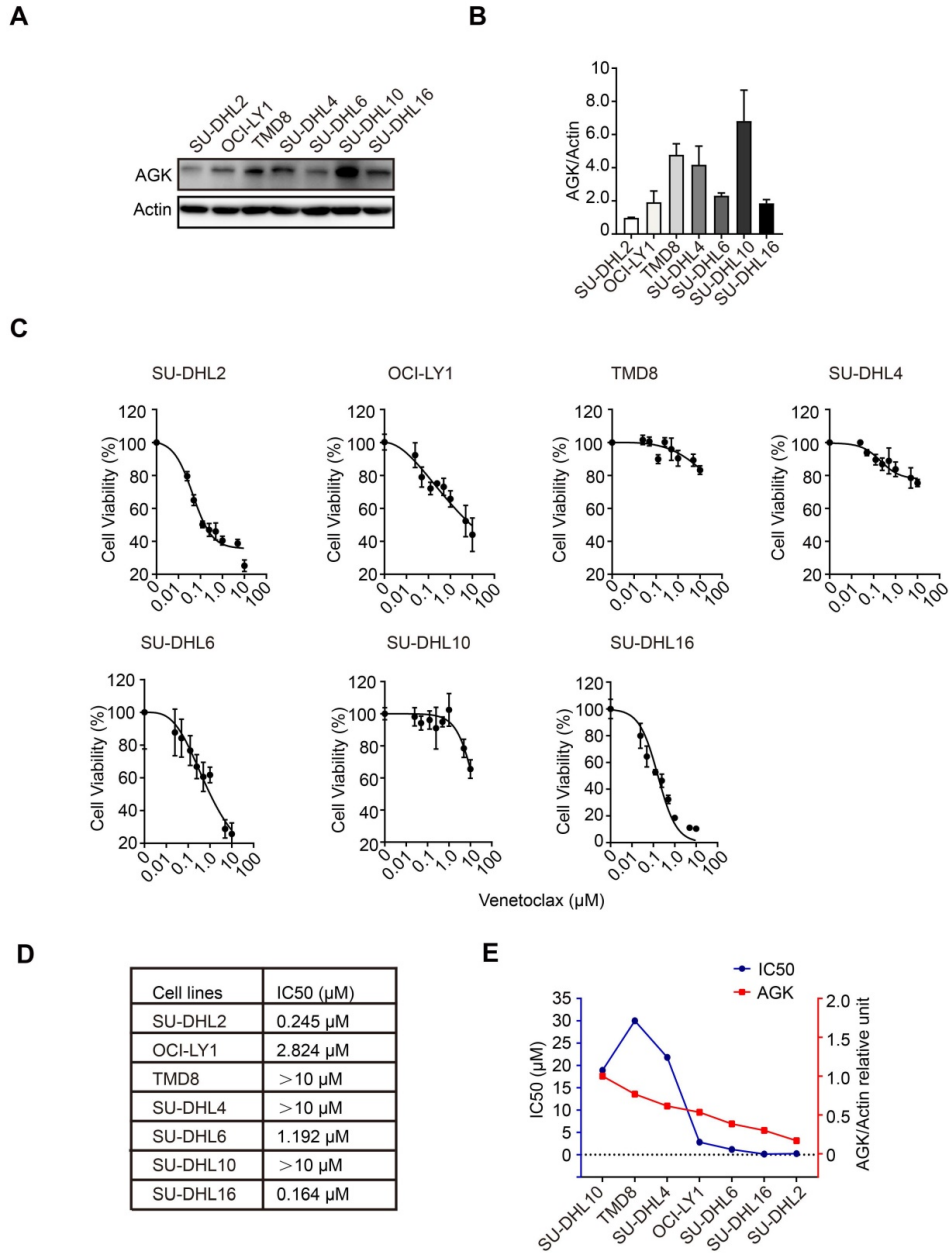
** AGK expression is inversely associate with the sensitivity to venetoclax in DLBCL cells. A**. AGK protein expression was determined using immunoblotting in SU-DHL2, OCI-LY1, TMD8, SU-DHL4, SU-DHL6, SU-DHL10, and SU-DHL16 DLBCL cell lines. **B.** The band intensities were quantified from three independent experiments. **C-D.** The IC50 of seven DLBCL cell lines to venetoclax was calculated with log (inhibitor) vs normalized response. **E.** The AGK expression and IC50 of venetoclax in seven DLBCL cell lines were presented by dual Y axis graph. The AGK/Actin ration in SU-DHL10 cells was set as 1. Data were expressed as mean ± SEM. The statistical significance was determined by One-way ANOVA (B, D).

To explore the functions of AGK in the regulation of sensitivity of DLBCL cells to venetoclax, we compared AGK expression in SU-DHL2, OCI-LY1, TMD8, SU-DHL4, SU-DHL6, SU-DHL10, and SU-DHL16 cells. The expression of AGK in SU-DHL2, OCI-LY1, SU-DHL6, and SU-DHL16 was low, while TMD8, SU-DHL4, and SU-DHL10 had higher expression of AGK (Figure 1A-B). Next, we compared the viability of these cell lines upon treatment of different concentrations of venetoclax using CCK-8 and calculated the IC50. We found that SU-DHL2, OCI-LY1, SU-DHL6, and SU-DHL16 were more sensitive to venetoclax (IC50, 0.245 µM, 2.824 µM, 1.192 µM, and 0.164 µM respectively), compared to TMD8, SU-DHL4, and SU-DHL10 (both >10 µM) (Figure 1C-D), suggesting that AGK expression was associated with resistance to venetoclax. In general, along with the descending expression of AGK expression, there was a trend of decreasing of IC50 to venetoclax except TMD8 (Figure 1E). Together, these data suggested that AGK may regulate the sensitivity of DLBCL cells to venetoclax.

### AGK knockdown sensitized DLBCL cells to venetoclax-induced apoptosis

**Figure 2:**
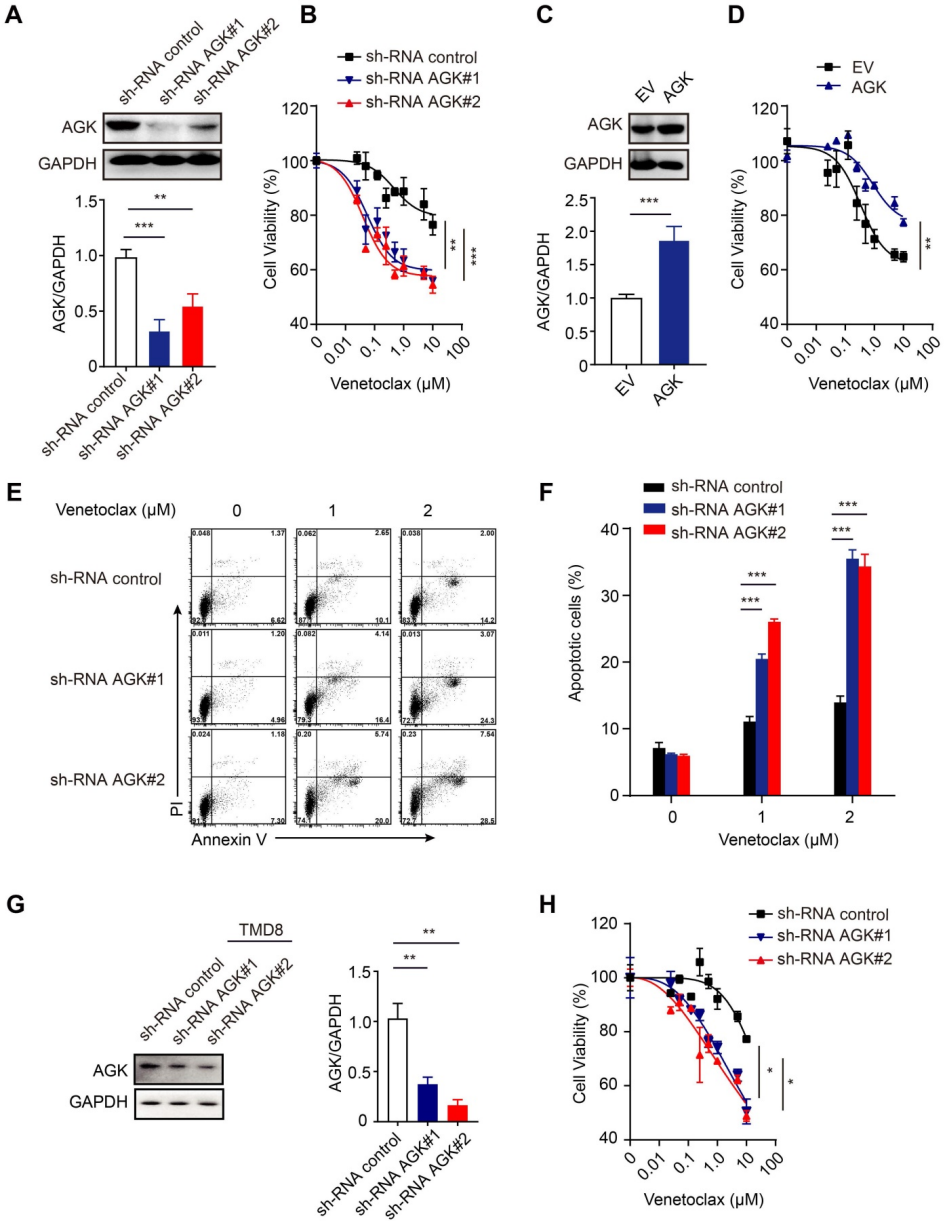
** AGK inhibits venetoclax-induced apoptosis in DLBCL cells. A.** SU-DHL4 cells were stably transduced with shRNA control lentiviral particles or shRNA AGK lentiviral particles. AGK protein expression was determined by immunoblotting and the band intensities were quantified from three independent experiments. **B.** AGK knockdown SU-DHL4 and control cell were treated with 0-10 μM venetoclax for 72 h, and cell viability was assessed by CCK-8 assays. **C.** SU-DHL4 cells were transduced with control retrovirus or AGK retrovirus and selected with 5 μg/mL puromycin for 14 days to generate stable cell lines. AGK protein expression was measured by immunoblotting and quantified from three independent experiments. **D.** Cell viability of control SU-DHL4 cells and AGK overexpression SU-DHL4 cell that were treated with venetoclax for 72 h. **E-F.** Apoptosis of control and AGK-knockdown SU-DHL4 cells was detected using Annexin Ⅴ/PI staining, and the percentage of apoptotic cells was quantified. **G.** TMD8 cells were stably transduced with shRNA control lentiviral particles or shRNA AGK lentiviral particles. AGK protein expression was determined by immunoblotting and the band intensities were quantified from three independent experiments.** H.** AGK knockdown TMD8 and control cell were treated with 0-10 μM venetoclax for 72 h, and cell viability was assessed by CCK-8 assays. Data were expressed as mean ± SEM from three independent experiments (A, C, F, G) or three independent experiments with duplicates (B, D, H), and analyzed for statistical significance by *t*-test (A, C, G) or Two-way ANOVA (B, D, F, H) (*P < 0.05; **P < 0.01; ***P < 0.001).

To further test the role of AGK in DLBCL, we generated AGK knockdown stable SU-DHL4 cells, whose expression of AGK was significantly lower, compared to the control cells (Figure 2A). Suppression of AGK expression significantly enhanced the sensitivity to venetoclax (Figure 2B). Conversely, stable overexpression of AGK in DLBCL cell lines rendered them more resistant to the effects of venetoclax (Figure 2C-D). Suppression of AGK expression rendered the cells more susceptible to venetoclax-induced apoptosis (Figure 2E-F). In addition, venetoclax induced cell apoptosis and caspase3 cleavage (Figure S2A), which could be abolished by Z-VAD-fmk, a pan caspase inhibitor (Figure S2B-C). We repeated the same experiments in AGK knockdown TMD8 cells and found that AGK knockdown significantly enhanced the sensitivity to venetoclax in TMD8 cells (Figure 2G-H).

Together, these data demonstrated that suppression of AGK expression enhances the sensitivity of DLBCL to venetoclax killing via enhanced apoptosis.

### AGK upregulates BCL-2 expression through AKT-FOXO1 axis in DLBCL cells

**Figure 3:**
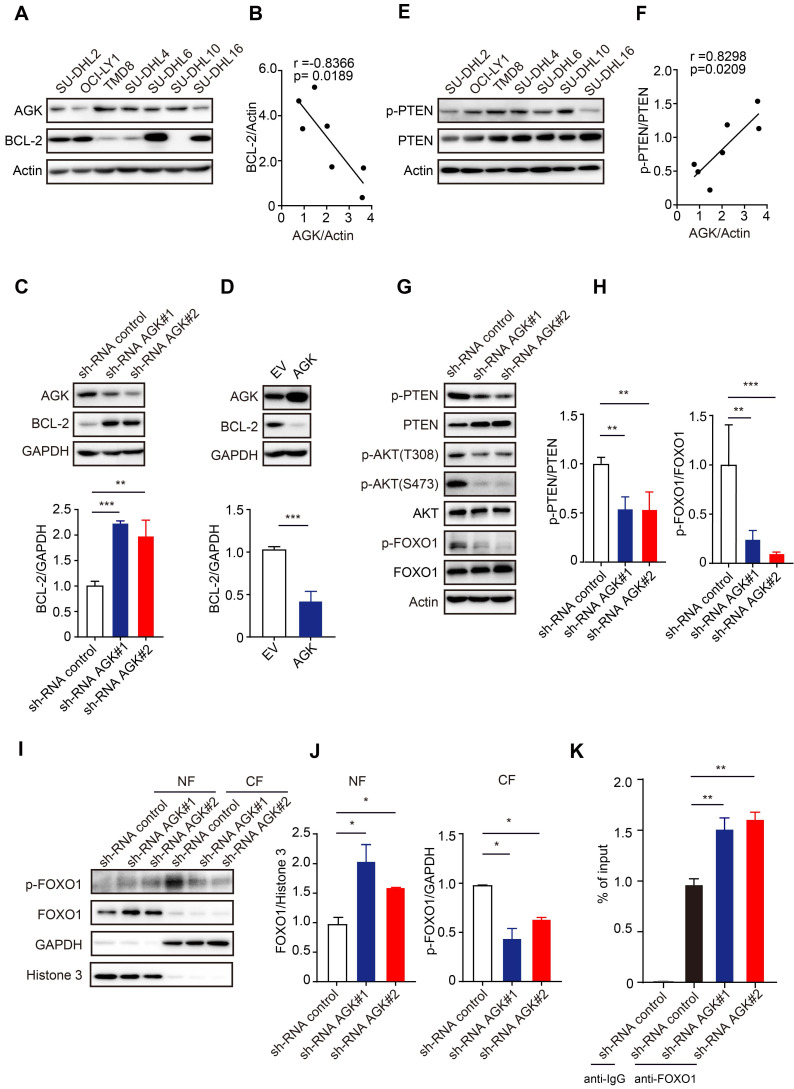
** AGK inhibits BCL-2 expression by phosphorylation PTEN and subsequent AKT-FOXO1 signaling. A.** The levels of AGK and BCL-2 were determined in SU-DHL2, OCI-LY1, TMD8, SU-DHL4, SU-DHL6, SU-DHL10, and SU-DHL16 DLBCL cell lines. **B.** Spearman correlation analysis of AGK and BCL-2 expression in DLBCL cell lines. **C.** AGK and BCL-2 expression was measured with immunoblotting and BCL-2 expression was quantified in shRNA control and shRNA AGK SU-DHL4 cells from three independent experiments. **D.** AGK and BCL-2 expression was measured in control and AGK overexpression SU-DHL4 cells, and BCL-2 expression was quantified from three independent experiments. **E.** The amounts of p-PTEN and PTEN in SU-DHL2, OCI-LY1, TMD8, SU-DHL4, SU-DHL6, SU-DHL10, and SU-DHL16 DLBCL cell lines were determined by immunoblotting. **F.** Spearman correlation analysis of AGK and PTEN phosphorylation levels in DLBCL cell lines. **G-H.** Phosphorylation levels of PTEN, AKT and FOXO1 were measured and quantified in control and AGK knockdown SU-DHL4 cells from three independent experiments. **I-J** The levels of FOXO1 and p-FOXO1 were measured in cytoplasmic and nuclear fractions (I) and the band intensities were quantified (J). **K.** The binding of FOXO1 to *BCL-2* promoter in SU-DHL4 was determined by chromatin immunoprecipitation analysis. Data were expressed as mean ± SEM from three independent experiments (A-J) or two independent experiments with triplicates (K), and analyzed for statistical significance by *t*-test. (*P < 0.05; **P < 0.01; ***P < 0.001).

Given the high selectivity of venetoclax to inhibit BCL-2, we wondered whether AGK regulates venetoclax sensitivity via regulation of BCL-2 expression. In contrast to AGK, BCL-2 expression in SU-DHL2, OCI-LY1, SU-DHL6, and SU-DHL16 was higher, while TMD8, SU-DHL4, and SU-DHL10 had lower expression of BCL-2 (Figure 3A). Spearman correlation analysis found that the expressions of AGK and BCL-2 were negatively correlated (Figure 3B). Compared to the control cells, cells stably transduced with AGK shRNA had significantly enhanced BCL-2 expression (Figure 3C). Similar results were obtained with TMD8 cells (Figure S3A). Conversely, overexpression of AGK in SU-DHL4 cells inhibited BCL-2 expression (Figure 3D). AGK can phosphorylate and inactivate PTEN 23. Consistent with this, we found that the phosphorylation levels of PTEN positively correlated with AGK expression (Figure 3E-F). AGK induced PTEN phosphorylation and inactivation, which was associated with enhanced phosphorylation of AKT at both Thr308 and Ser473, and FOXO1 at Thr24, a known target of AKT in both SU-DHL4 (Figure 3G-H) and TMD8 cells (Figure S3B-C). Dephosphorylation of FOXO1 was associated with reduced cytoplasmic levels of FOXO1 and enhanced nuclear localization (Figure 3I-J, and Figure S3D-E). Conversely, overexpression of AGK in SU-DHL4 cells enhanced the phosphorylation of AKT and FOXO1, and reduced nuclear localization of FOXO1 (Figure S3F-G). Furthermore, knockdown of AGK enhanced FOXO1 binding to the *BCL-2* promoter (Figure 3K, and Figure S3H).

Taken together, these data demonstrated that AGK inhibits BCL-2 expression via phosphorylation of PTEN, which subsequently enhanced phosphorylation of AKT and reduced FOXO1 transcriptional activity.

### Suppression of AGK expression enhanced the sensitivity of SU-DHL4 to venetoclax in a xenograft tumor model

**Figure 4:**
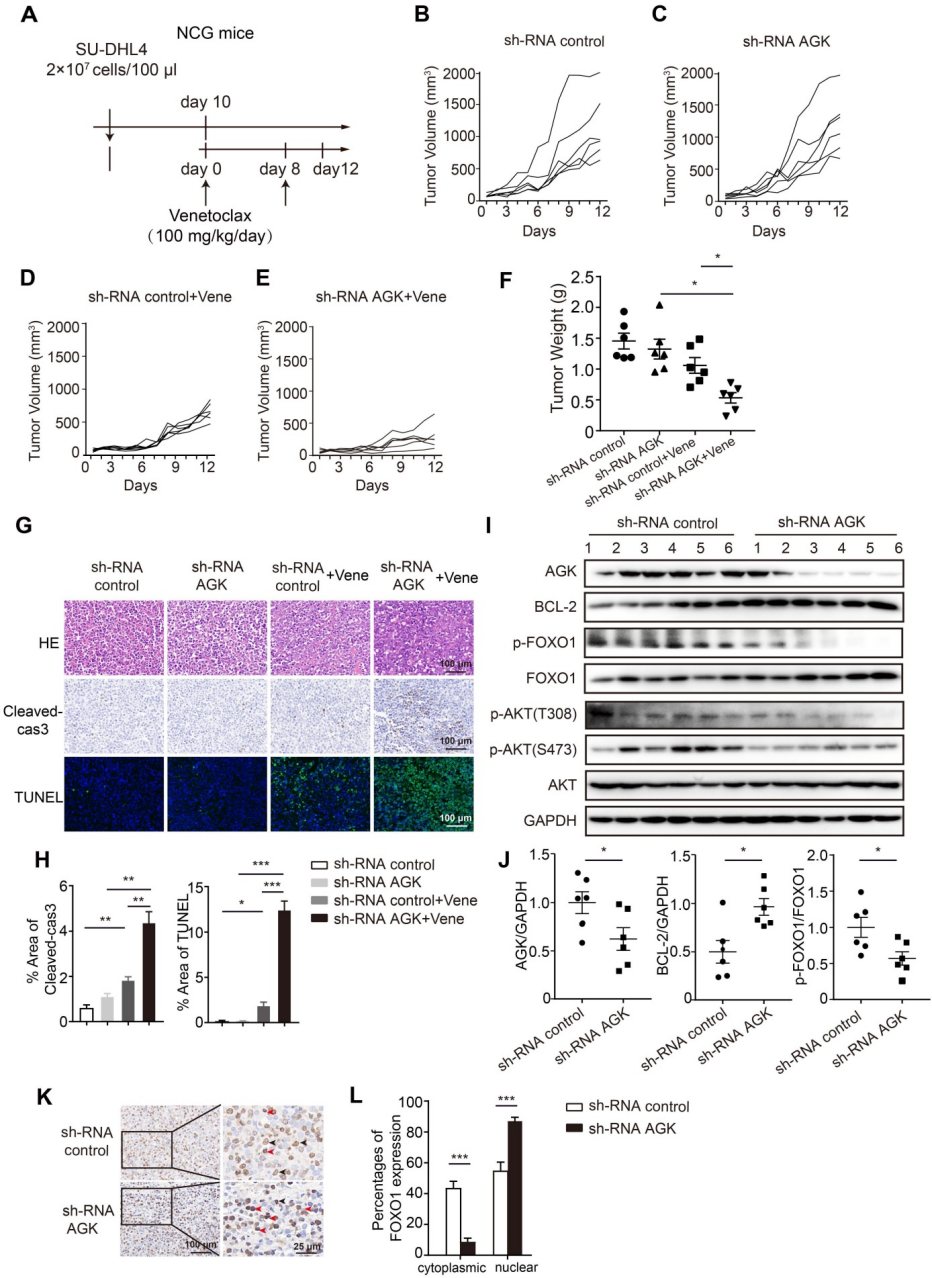
**Knockdown of AGK augments venetoclax efficacy *in vivo*. A.** Schematic diagram of xenograft tumor model and venetoclax treatment strategy. **B-E.** Tumor growth curves of mice receiving shRNA control, shRNA AGK#1 cells with or without venetoclax treatment (n = 6, each group). **F.** Tumor weights of shRNA control and shRNA AGK#1 groups with or without venetoclax treatment (n = 6, each group). **G-H.** H&E staining, cleaved-caspase3 and TUNEL staining of shRNA control and shRNA AGK groups with or without venetoclax treatment, and the percentages of positive area of cleaved-caspase3 and TUNEL were quantified. **I-J.** The levels of AGK, BCL-2, p-FOXO1, FOXO1, p-AKT and AKT were measured (I) and quantified (J) in the tumor tissues isolated from shRNA control and shRNA AGK groups (n = 6, each group). **K-L.** FOXO1 staining of shRNA control and shRNA AGK group tumor tissues (K) and the percentages of FOXO1 expression in cytoplasmic and nuclear were quantified (L). Scale bar, 100 μm or 25 μm. Data were expressed as mean ± SEM and analyzed for statistical significance by One-way ANOVA (F, H, L) or *t*-test (J) (*P < 0.05; **P < 0.01; ***P < 0.001).

To determine the relevance of AGK-mediated venetoclax sensitivity of DLBCL cells *in vivo*, we employed a widely used xenograft tumor model. Immunodeficient NCG mice were subcutaneously injected with control SU-DHL4 cells or cells stably transduced with AGK shRNA. Venetoclax or vehicle solution was *i.p* administrated as schematic depicted (Figure 4A). Consistent with *in vitro* data, there was no significant difference in tumor volumes between the shRNA control and shRNA AGK groups (Figure 4B-C). However, upon venetoclax treatment, tumors derived from the AGK knockdown group grew more slowly, compared with the shRNA control group (Figure 4D-F, and Figure S4A). The tumors in venetoclax treatment groups had higher numbers of apoptotic cells compared to the control group, which was further enhanced in shRNA AGK group, as measured by caspase3 cleavage and TUNEL staining (Figure 4G-H). Upon treatment with venetoclax, the numbers of Ki-67 and α-SMA positive cells in the shRNA AGK group were reduced, compared to the control group (Figure S4B-C). In the tumor tissues of AGK knockdown group, we found that the expression of BCL-2 was upregulated, but the phosphorylation of FOXO1 and AKT was significantly decreased (Figure 4I-J). Compared to the control group, the percentages of cytoplasmic FOXO1 positive cells were reduced in shRNA AGK group tumor tissues, while the nuclear localizations of FOXO1 were significantly enhanced in shRNA AGK group (Figure 4K-L).

Taken together, these data demonstrated that inhibition of AGK renders DLBCL cells more sensitive to venetoclax-induced apoptosis *in vivo*.

### Inhibition of AGK augments venetoclax sensitivity via enhanced FOXO1-mediated BCL-2 expression

**Figure 5:**
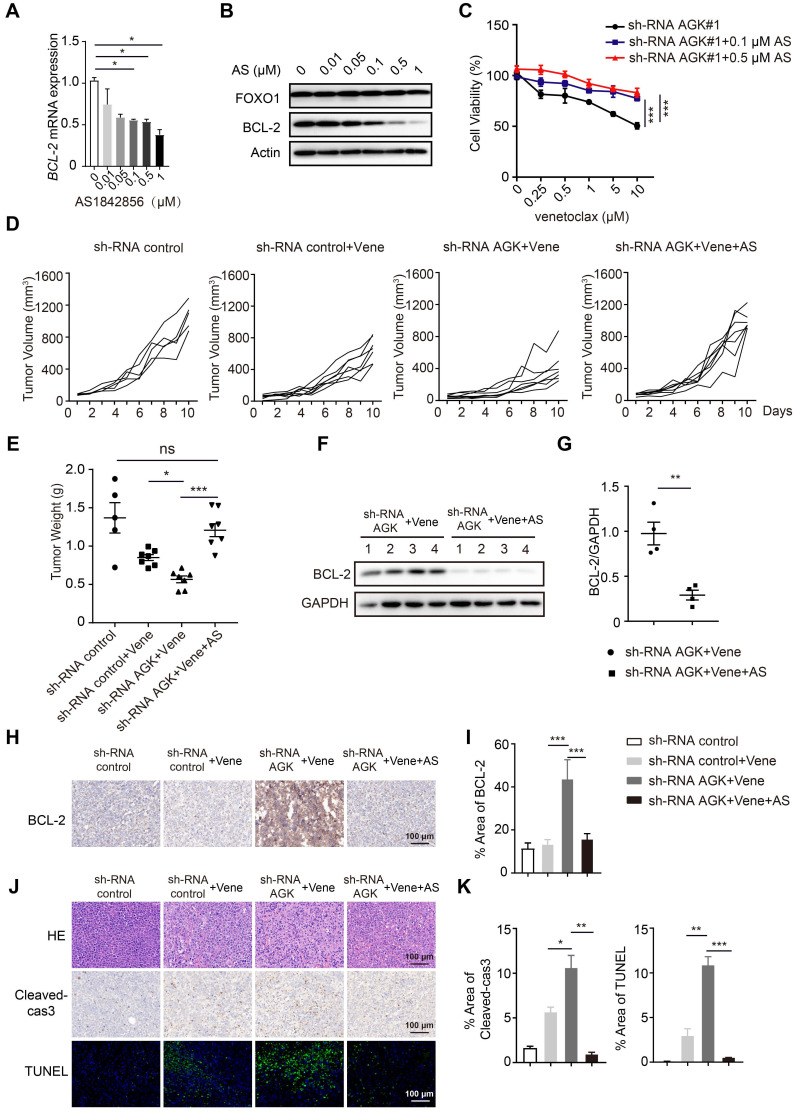
** Inhibition of FOXO1 abolished enhanced sensitivity to venetoclax of AGK knockdown. A-B.** The mRNA and protein expression of BCL-2 were measured in SU-DHL4 shRNA AGK#1 cells upon treatment of AS1842856 for 24 h. The band intensities were quantified from three independent experiments. **C.** Cell viability of SU-DHL4 shRNA AGK#1 cells with or without AS1842856 treatment was determined. The data showed mean ± SEM from three independent experiments with duplicates.** D-E.** NCG mice were implanted with shRNA control cells or shRNA AGK#1 cells treated with venetoclax or venetoclax plus AS1842856. At day 10 after *i.p* administration, mice were sacrificed. Tumor volume change curves were recorded (D) and tumor weights (E) were presented (n ≥ 5, each group). **F-G.** The levels of BCL-2 were measured (E) and quantified (F) in the tumor tissues isolated from shRNA AGK groups treated with venetoclax alone and co-treated with venetoclax and AS1842856 (n = 4, each group).** H-I.** Immunohistochemistry staining of BCL-2 in the four groups as shown, and the percentages of positive areas of BCL-2 were quantified.** J-K.** H&E staining and cleaved-caspase3 and TUNEL staining of the four groups as shown, and the percentages of positive areas of cleaved-caspase3 and TUNEL were quantified. Scale bar, 100 μm. Data were expressed as mean ± SEM and analyzed for statistical significance by One-way ANOVA (A, C, E, I, K) or *t*-test (G). (*P < 0.05; **P < 0.01; ***P < 0.001; ns, not significant).

Next we asked whether blockade of FOXO1 transcriptional activity could reverse the AGK-mediated regulation of sensitivity to venetoclax. AS1842856, a FOXO1 inhibitor, binds and inhibits FOXO1 transcriptional activity 24. We found that AS1842856 inhibited BCL-2 expression at both mRNA and protein levels in a dose-dependent manner and reversed the sensitivity of DLBCL to venetoclax in shRNA AGK SU-DHL4 cells (Figure 5A-C). Consistent with these results *in vitro*, AS1842856 treatment completely abolished the enhanced sensitivity to venetoclax on tumor volume and tumor weight in the AGK knowndown group (Figure 5D-E, and Figure S5A). Compared to shRNA control group, BCL-2 expression was significantly increased in the tumor tissues from shRNA AGK group, and AS1842856 treatment abolished the enhanced expression of BCL-2 (Figure 5F-I). Similarly, Ki67 and α-SMA staining data showed that AS1842856 treatment reversed the potentiated cell killing of venetoclax in the AGK knockdown group (Figure S5B-C). Similar patterns were found for cleaved-caspase3 and TUNEL staining (Figure 5J-K). Together, these data demonstrated that inhibition of AGK sensitizes DLBCL to venetoclax via enhanced FOXO1-mediated BCL-2 expression.

### BCL-2 expression is inversely correlated with the level of AGK in DLBCL patients

**Figure 6:**
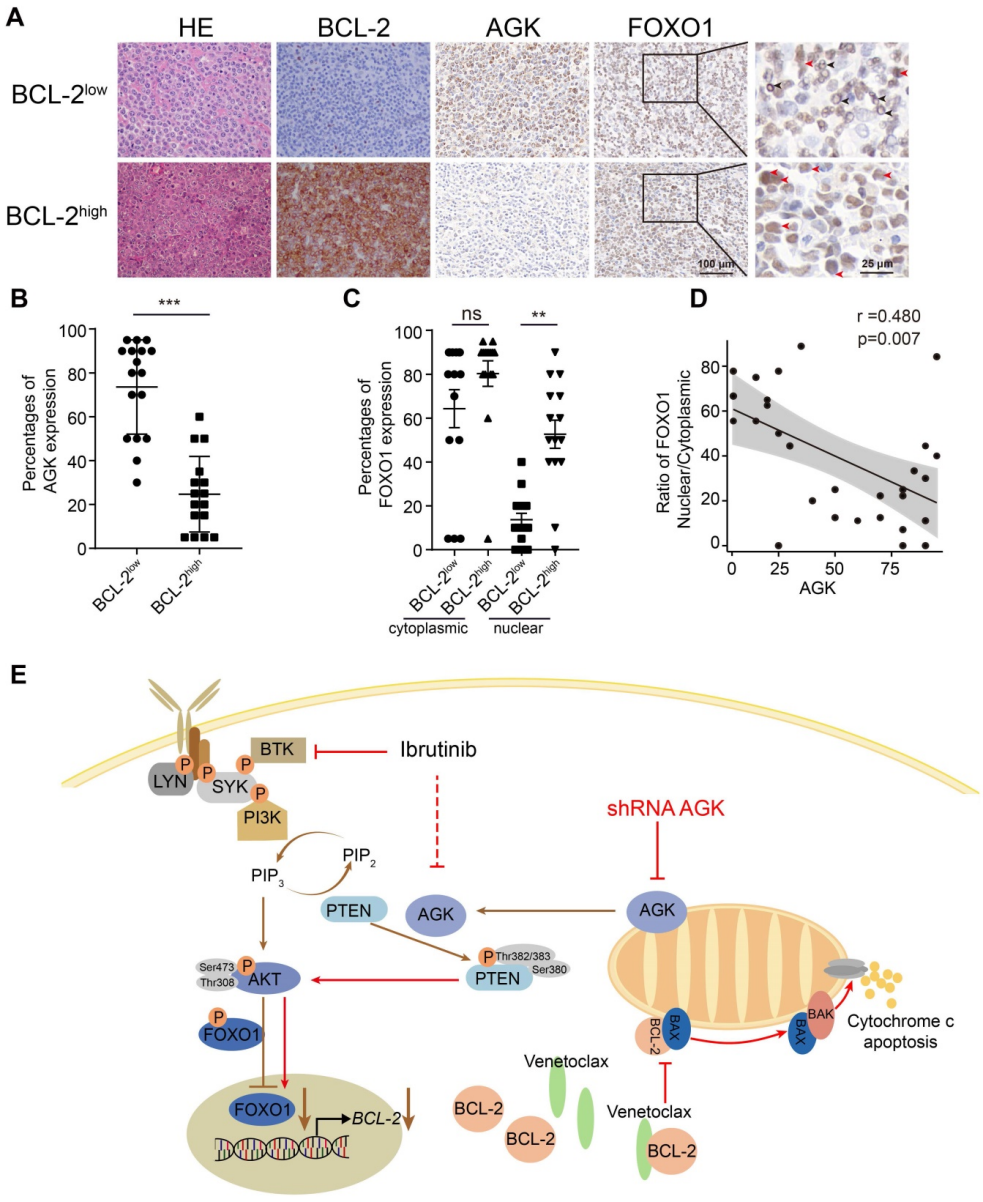
** The expression of AGK is inversely associated with BCL-2 expression in DLBCL patients. A.** H&E staining, BCL-2, AGK and FOXO1 staining of BCL-2^low^ and BCL-2^high^ DLBCL tissue samples. n = 33, scale bar, 100 μm or 25 μm. Black arrow: cytoplasmic FOXO1; Red arrow: nuclear FOXO1. **B.** Quantitation of the percentages of AGK positive cell in BCL-2^low^ (n = 17) and BCL-2^high^ (n = 16) DLBCL paraffin sections. **C.** Quantitation of the ratios of nuclear and cytoplasmic FOXO1 in BCL-2^low^ (n = 15) and BCL-2^high^ (n = 14) DLBCL paraffin sections. **D.** Spearman correlation analysis of the ratios of nuclear versus cytoplasmic FOXO1 with the expression of AGK. **E.** Schematic figure of the regulation of AGK on venetoclax sensitivity. In DLBCL cells, AGK phosphorylates and inactivates PTEN, leading to enhanced phosphorylation of AKT and FOXO1 and reduced FOXO1 nuclear translocation and its target gene *BCL-2* expression. AGK knockdown could reverse the effect of AGK-PTEN-FOXO1-BCL-2 axis, upregulate the expression of BCL-2 and increase the sensibility to venetoclax-induced apoptosis. Ibrutinib inhibits the expression of AGK in DLBCL cells. Data were expressed as mean ± SEM and analyzed for statistical significance by* t* test (B, D). (**P < 0.01; ***P < 0.001; ns, not significant).

To investigate whether the expression of AGK could be a biomarker for predicting the response to venetoclax in DLBCL patients, we performed H&E and BCL-2 immunohistochemical staining for human DLBCL primary tumor tissues of 33 individual patients. The molecular subtypes of the BCL-2 translocation status were shown in the supplementary data (Table S1). The samples were divided into BCL-2^high^ and BCL-2^low^ groups based on whether the percentages of BCL-2 positive areas were more than 5%. Then we compared the levels of both AGK and FOXO1 in these two groups (Figure 6A). The expression of AGK were significantly higher in the BCL-2^low^ group, compared to the BCL-2^high^ group (Figure 6B). In line with the results *in vitro*, the nuclear localizations of FOXO1 were significantly higher in the BCL-2^high^ group (Figure 6C). AGK expression was negatively correlated with the nuclear localizations of FOXO1 (Figure 6D). Together, these results demonstrated that BCL-2 expression is inversely regulated by AGK in DLBCL patients, indicating that AGK not only can serve as a biomarker for venetoclax response but also combination of targeting AGK with venetoclax could provide benefit for the treatment of aggressive DLBCL.

It has been shown that ibrutinib can sensitize DLBCL cells to venetoclax killing both *in vitro* and *in vivo* 25. Consistent with this, SU-DHL4 cells could be sensitized to venetoclax by ibrutinib (Figure S6A). To investigate whether AGK is involved in this process, we measured the expression of AGK upon treatment of ibrutinib. Ibrutinib inhibited AGK expression in SU-DHL4 cells (Figure S6B-C). To test whether the downregulation of AGK mediates the ibrutinib-induced sensitization to venetoclax, we forced AGK expression in SU-DHL4 cells. Overexpression of AGK significantly alleviated the cytotoxic effect induced by the combination of ibrutinib and venetoclax (Figure S6D). Together, these data demonstrated that AGK mediates the ibrutinib-induced sensitization of DLBCL cells to venetoclax.

The regulation of AGK on venetoclax sensitivity could be summarized as schematic figure (Figure 6E).

## Discussion

Despite the success of venetoclax in the treatment of CLL and AML, there is little preclinical and clinical data to suggest a benefit in DLBCL. However, there may be a subset of patients that could benefit from this therapy. Identification of novel biomarkers for predicting the treatment response of venetoclax would be essential for these patients. Furthermore, this knowledge could be used to generate combinatory regimens to enhance the efficacy of venetoclax in the wider population of DLBCL patients. In this study, we identified that knockdown of AGK in DLBCL sensitizes the cells to venetoclax-induced apoptosis. AGK phosphorylates and inactivates PTEN, enhancing PI3K and AKT activation and subsequently suppressing FOXO1-mediated BCL-2 expression.

Although initially identified as a lipid kinase, AGK has been shown to phosphorylate GSK3β at Ser9 and PTEN at Ser380, Thr382, and Thr383 residues to inactivate them, leading to enhanced PI3K-AKT activation in renal cell carcinoma cells (RCC) and CD8^+^ T cells respectively 23, 26. In our study, suppression of AGK inhibited PTEN phosphorylation in DLBCL cells. However, knockdown of AGK did not affect cell proliferation and migration (Data not shown). A previous study reported that AGK can suppress FOXO1 transcriptional activity in breast cancer cells 27, similar to our study. The abolishing of sensitization to venetoclax of downregulation of AGK by FOXO1 inhibitor AS1842856 *in vivo* highlights the importance of AGK-PTEN-FOXO1-BCL-2 axis in regulation of venetoclax response. FOXO1 plays essential roles in regulation of stage transitions during B cell development and GC development 28, 29. FOXO1 is recurrently targeted by specific mutations in 9% DLBCL patients. These mutations may induce resistance to PI3K-mediated phosphorylation and inactivation in the cytoplasm 30. In addition, it can mediate BCL-6-mediated oncogenic activity 29. FOXO1 can be phosphorylated by GSK-3β and it has been reported that GSK-3β inhibitor 9-ING-41 treatment sensitizes SU-DHL4 and KPUM-UH1 cells to venetoclax 31, which highlights the complex regulation of FOXO1 in DLBCL.

The role of BCL-2 expression in predicting the response of DLBCL to venetoclax is complex. Initially it has been showed that the level of BCL-2 expression measured by IHC did not correlate to the clinical response in a phase I study 32. However, in a phase Ⅱ study CAVALLI of venetoclax plus R-CHOP as first-line treatment for DLBCL, the addition of venetoclax demonstrated improved efficacy, particularly in high-risk BCL-2^+^ patient subgroup 33. In line with this, Pham et* al.* found that the presence of BCL-2 primarily dictates the cellular sensitivity to venetoclax in aggressive DLBCL cell lines, as well as primary aggressive cells and *in vivo* patient-derived tumor xenograft models 34, suggesting a possible stochiometric effect of venetoclax by BCL-2 expression. Our data found a negative association of AGK with BCL-2, which was linked to sensitivity to venetoclax-induced apoptosis. The discrepancy of reliance of BCL-2 expression for venetoclax sensitivity could be due to the heterogeneity of DLBCL cells, whose intrinsic and extrinsic apoptosis pathways were regulated by complex networks of apoptotic and anti-apoptotic proteins such as BCL-XL and MCL-1 35. Furthermore, BCL-2^+^ DLBCL cells may bear BCL-2 mutations i.e*.* Gly101Val and Phe104Leu/Cys, which render them resistant to venetoclax 25, 36. In our study, even in SU-DHL4 cells that harbors BCL-2 translocation, downregulation of AGK enhanced FOXO1-mediated BCL-2 expression, suggesting the general regulation of BCL-2 by AGK. However, we did not generate venetoclax-resistant cell lines by long term exposure of cells to low doses of venetoclax, which is a limitation of this study.

Multiple strategies to enhance the venetoclax efficacy for lymphomas have been documented 9, 14. For example, combinations of venetoclax with either a pan PI3K inhibitor copanlisib or the BTK inhibitor ibrutinib were able to enhance the venetoclax efficacy for relapse/refractory DLBCL in clinical studies. Short or long exposure of venetoclax has been shown to downregulate PTEN expression, resulting in enhanced PI3K-AKT activation 34, providing the rationale for targeting both PI3K-AKT and BCL-2 simultaneously. We found that ibrutinib inhibited AGK expression in SU-DHL4 cells, although the mechanism is not clear. Overexpression of AGK abolished the synergistic effect of ibrutinib with venetoclax, suggesting a critical role for this kinase.

Currently there is no AGK inhibitor that has been developed. Targeting AGK may represent a new strategy to enhance the efficacy of venetoclax in DLBCL. Our data demonstrate that targeting AGK together with venetoclax may provide benefit for DLBCL patients, especially for the high-grade aggressive double hit lymphoma or double expressor lymphoma.

## Abrreviations

DLBCL: Diffuse large B-cell lymphoma; R-CHOP: Rituximab plus cyclophosphamide, doxorubicin, vincristine, and prednisone; NHL: Non-hodgins lymphoma; BCL-2: B-cell leukemia/lymphoma-2; CLL: Chronic lymphocytic leukemia; SLL: Small lymphocytic lymphoma; AML: Acute myeloid leukemia; ORR: Overall response rate; AGK: Acylglycerol kinase; CCK-8: Cell Counting Kit-8; CHIP: Chromatin immunoprecipitation.

## Supplementary Material

Supplementary figures.

## References

[1] Bojarczuk K, Wienand K, Ryan JA, Chen L, Villalobos-Ortiz M, Mandato E (2019). Targeted inhibition of PI3Kα/δ is synergistic with BCL-2 blockade in genetically defined subtypes of DLBCL. Blood.

[2] Schmitz R, Wright GW, Huang DW, Johnson CA, Phelan JD, Wang JQ (2018). Genetics and Pathogenesis of Diffuse Large B-Cell Lymphoma. N Engl J Med.

[3] Coiffier B, Lepage E, Briere J, Herbrecht R, Tilly H, Bouabdallah R (2002). CHOP chemotherapy plus rituximab compared with CHOP alone in elderly patients with diffuse large-B-cell lymphoma. N Engl J Med.

[4] Vitolo U, Novo M (2021). Bcl-2 inhibition in DLBCL: "the times they are a-changing"?. Blood.

[5] Czabotar PE, Lessene G, Strasser A, Adams JM (2014). Control of apoptosis by the BCL-2 protein family: implications for physiology and therapy. Nat Rev Mol Cell Biol.

[6] Ryan CE, Davids MS (2019). BCL-2 Inhibitors, Present and Future. Cancer J.

[7] Johnson NA, Slack GW, Savage KJ, Connors JM, Ben-Neriah S, Rogic S (2012). Concurrent expression of MYC and BCL2 in diffuse large B-cell lymphoma treated with rituximab plus cyclophosphamide, doxorubicin, vincristine, and prednisone. J Clin Oncol.

[8] Valentin R, Grabow S, Davids MS (2018). The rise of apoptosis: targeting apoptosis in hematologic malignancies. Blood.

[9] Davids MS (2017). Targeting BCL-2 in B-cell lymphomas. Blood.

[10] Souers AJ, Leverson JD, Boghaert ER, Ackler SL, Catron ND, Chen J (2013). ABT-199, a potent and selective BCL-2 inhibitor, achieves antitumor activity while sparing platelets. Nat Med.

[11] Pollyea DA, Pratz K, Letai A, Jonas BA, Wei AH, Pullarkat V (2021). Venetoclax with azacitidine or decitabine in patients with newly diagnosed acute myeloid leukemia: Long term follow-up from a phase 1b study. Am J Hematol.

[12] Pollyea DA, Amaya M, Strati P, Konopleva MY (2019). Venetoclax for AML: changing the treatment paradigm. Blood Adv.

[13] Davids MS, Roberts AW, Seymour JF, Pagel JM, Kahl BS, Wierda WG (2017). Phase I First-in-Human Study of Venetoclax in Patients With Relapsed or Refractory Non-Hodgkin Lymphoma. J Clin Oncol.

[14] Kapoor I, Bodo J, Hill BT, Hsi ED, Almasan A (2020). Targeting BCL-2 in B-cell malignancies and overcoming therapeutic resistance. Cell Death Dis.

[15] Salah HT, DiNardo CD, Konopleva M, Khoury JD (2021). Potential Biomarkers for Treatment Response to the BCL-2 Inhibitor Venetoclax: State of the Art and Future Directions. Cancers (Basel).

[16] Guièze R, Liu VM, Rosebrock D, Jourdain AA, Hernández-Sánchez M, Martinez Zurita A (2019). Mitochondrial Reprogramming Underlies Resistance to BCL-2 Inhibition in Lymphoid Malignancies. Cancer Cell.

[17] Bektas M, Payne SG, Liu H, Goparaju S, Milstien S, Spiegel S (2005). A novel acylglycerol kinase that produces lysophosphatidic acid modulates cross talk with EGFR in prostate cancer cells. J Cell Biol.

[18] Mayr JA, Haack TB, Graf E, Zimmermann FA, Wieland T, Haberberger B (2012). Lack of the mitochondrial protein acylglycerol kinase causes Sengers syndrome. Am J Hum Genet.

[19] Vukotic M, Nolte H, König T, Saita S, Ananjew M, Krüger M (2017). Acylglycerol Kinase Mutated in Sengers Syndrome Is a Subunit of the TIM22 Protein Translocase in Mitochondria. Mol Cell.

[20] Chu B, Hong Z, Zheng X (2021). Acylglycerol Kinase-Targeted Therapies in Oncology. Front Cell Dev Biol.

[21] Jiang H, Yu Z, Ding N, Yang M, Zhang L, Fan X (2020). The role of AGK in thrombocytopoiesis and possible therapeutic strategies. Blood.

[22] Zhao C, Chen HY, Zhao F, Feng HJ, Su JP Acylglycerol kinase promotes paclitaxel resistance in nasopharyngeal carcinoma cells by regulating FOXM1 via the JAK2/STAT3 pathway. Cytokine. 2021: 155595.

[23] Hu Z, Qu G, Yu X, Jiang H, Teng XL, Ding L (2019). Acylglycerol Kinase Maintains Metabolic State and Immune Responses of CD8(+) T Cells. Cell Metab.

[24] Wang F, Demir S, Gehringer F, Osswald CD, Seyfried F, Enzenmüller S (2018). Tight regulation of FOXO1 is essential for maintenance of B-cell precursor acute lymphoblastic leukemia. Blood.

[25] Kuo HP, Ezell SA, Schweighofer KJ, Cheung LWK, Hsieh S, Apatira M (2017). Combination of Ibrutinib and ABT-199 in Diffuse Large B-Cell Lymphoma and Follicular Lymphoma. Mol Cancer Ther.

[26] Zhu Q, Zhong AL, Hu H, Zhao JJ, Weng DS, Tang Y (2020). Acylglycerol kinase promotes tumour growth and metastasis via activating the PI3K/AKT/GSK3β signalling pathway in renal cell carcinoma. J Hematol Oncol.

[27] Wang X, Lin C, Zhao X, Liu A, Zhu J, Li X (2014). Acylglycerol kinase promotes cell proliferation and tumorigenicity in breast cancer via suppression of the FOXO1 transcription factor. Mol Cancer.

[28] Inoue T, Shinnakasu R, Ise W, Kawai C, Egawa T, Kurosaki T (2017). The transcription factor Foxo1 controls germinal center B cell proliferation in response to T cell help. J Exp Med.

[29] Dominguez-Sola D, Kung J, Holmes AB, Wells VA, Mo T, Basso K (2015). The FOXO1 Transcription Factor Instructs the Germinal Center Dark Zone Program. Immunity.

[30] Trinh DL, Scott DW, Morin RD, Mendez-Lago M, An J, Jones SJ (2013). Analysis of FOXO1 mutations in diffuse large B-cell lymphoma. Blood.

[31] Karmali R, Chukkapalli V, Gordon LI, Borgia JA, Ugolkov A, Mazar AP (2017). GSK-3β inhibitor, 9-ING-41, reduces cell viability and halts proliferation of B-cell lymphoma cell lines as a single agent and in combination with novel agents. Oncotarget.

[32] Herrera AF, Mei M, Low L, Kim HT, Griffin GK, Song JY (2017). Relapsed or Refractory Double-Expressor and Double-Hit Lymphomas Have Inferior Progression-Free Survival After Autologous Stem-Cell Transplantation. J Clin Oncol.

[33] Morschhauser F, Feugier P, Flinn IW, Gasiorowski R, Greil R, Illés Á (2021). A phase 2 study of venetoclax plus R-CHOP as first-line treatment for patients with diffuse large B-cell lymphoma. Blood.

[34] Pham LV, Huang S, Zhang H, Zhang J, Bell T, Zhou S (2018). Strategic Therapeutic Targeting to Overcome Venetoclax Resistance in Aggressive B-cell Lymphomas. Clin Cancer Res.

[35] Deng J, Carlson N, Takeyama K, Dal Cin P, Shipp M, Letai A (2007). BH3 profiling identifies three distinct classes of apoptotic blocks to predict response to ABT-737 and conventional chemotherapeutic agents. Cancer Cell.

[36] Tahir SK, Smith ML, Hessler P, Rapp LR, Idler KB, Park CH (2017). Potential mechanisms of resistance to venetoclax and strategies to circumvent it. BMC Cancer.

