# Supplementary material for: Inhibition of acylglycerol kinase sensitizes DLBCL to venetoclax via upregulation of FOXO1-mediated BCL-2 expression
Source: Theranostics. 2022 Jul 18;12(12):5537–50. doi: 10.7150/thno.72786 (PMC9330532; doi:10.7150/thno.72786)
Supplement: Supplementary file 1 — Supplementary figures. [file thnov12p5537s1.pdf]

Figure S1. AGK is linked to venetoclax sensitivity of DLBCL cells. (A) The expression profiling of two parental cell lines and two venetoclax-resistant cell lines were analyzed using gene ontology (GO) analysis from the Gene Expression Omnibus (GEO) database under accession number GSE128563 and enriched pathways related to mitochondrial structure and function and lipid metabolism were identified. X-axis: percentage of gene involved, Y-axis: functional pathways. (B) Venn diagram of genes in enriched pathways. Differentially expressed genes with a cutoff of $log_2FC > 1$ were selected to identify enriched biological processes. Enriched pathways were selected by a cutoff of p value < 0.05.

Figure S2. Pan apoptosis inhibitor Z-VAD-fmk blocks ventoclax-induced cell death of SU-DHL4 cells. (A) The levels of cleaved-caspase3 were determined in shRNA control cells, or shRNA AGK1 and shRNA AGK2 SU-DHL4 cells after 48 h of the treatment of varying amounts of venetoclax. (B-C) SU-DHL4 cells were pretreated with different amounts of Z-VAD-fmk, followed with treatment of venetoclax (5 μM) for 48 h, the amounts of cleaved-caspase 3 were determined by immunoblotting (B) and apoptosis was measured and quantified by Annexin V/PI staining (C). All experiments were repeated three times with similar results. Data were expressed as mean ± SEM and analyzed for statistical significance by *t* test (**P* < 0.05; ****P* < 0.001).

Figure S3. AGK promotes phosphorylation of AKT and FOXO1 and inhibits FOXO1 nuclear translocation and BCL-2 expression. (A) AGK and BCL-2 expression was measured with immunoblotting and BCL-2 expression was quantified in shRNA control and shRNA AGK TMD8 cells from three independent experiments. (B-C) Phosphorylation levels of PTEN, AKT and FOXO1 were measured and quantified in control and AGK knockdown TMD8 cells from three independent experiments. (D-E) The levels of FOXO1 and p-FOXO1 in cytoplasmic and nuclear fractions were measured and the band intensities were quantified from three independent experiments. (F) The levels of p-AKT and p-FOXO1 were determined in SU-DHL4 stable cells transduced with empty retrovirus or AGK retrovirus. (G) Cytoplasmic and nuclear fractions were isolated from SU-DHL4 stable cells transduced with empty retrovirus or AGK retrovirus. The amounts of p-FOXO1 and FOXO1 were determined by immunoblotting. All experiments were repeated three times and one representative experiment was shown. Data were expressed as mean ± SEM and analyzed for statistical significance by *t* test (*$P < 0.05$; **$P < 0.01$; ***$P < 0.001$).

Figure S4. AGK knockdown enhanced venetoclax efficacy for DLBCL in vivo. (A) Tumor volumes of mice receiving shRNA control, shRNA AGK#1 cells with or without venetoclax treatment (n=6, each group). (B) Ki67 and α-SMA staining of tumor tissues isolated from NCG mice implanted with shRNA control SU-DHL4 cells, shRNA SU-DHL4 cells treated with or without venetoclax. (C) The percentages of positive areas of Ki67 and α-SMA were quantified, scale bar, 100 μm, Data were pooled in duplicate view from three mice per group. One representative image was shown. Data were expressed as mean ± SEM and analyzed for statistical significance by One-way ANOVA (*$P < 0.05$; **$P < 0.01$; ***$P < 0.001$; ns, not significant).

Figure S5. AS1842856 abolishes AGK knockdown-induced sensitization effect to venetoclax. (A) Tumor volumes of mice receiving shRNA control cells or shRNA AGK#1 cells treated with venetoclax or venetoclax plus AS1842856. (B) NCG mice were implanted with shRNA control cells or shRNA AGK cells treated with venetoclax or venetoclax plus AS1842856. Ki67 and α-SMA staining of tumor tissues isolated from shRNA control, shRNA control + venetoclax, shRNA AGK + venetoclax and shRNA AGK + venetoclax + AS1842856 four groups. (C) The percentages of positive areas of Ki67 and α-SMA were quantified, scale bar, 100 μm, Data were pooled in duplicate view from three mice per group. Representative images were shown. Data were expressed as mean ± SEM and analyzed for statistical significance by One-way ANOVA (*P < 0.05; **P < 0.01; ***P < 0.001).

Figure S6. AGK mediates the synergistic effect of ibrutinib with venetoclax in DLBCL cells. (A) Cell viability of SU-DHL4 cells after 72 h exposure to various amounts of ibrutinib and venetoclax. (B-C) AGK expression in SU-DHL4 cells was determined by immunoblotting (B) and the band intensities were quantified (C) after 24 h exposure to various amounts of ibrutinib. The experiments were repeated for three times. (D) Cell viability of SU-DHL4 stable cells transduced with empty retrovirus or AGK retrovirus that were treated with varying amounts of ibrutinib and venetoclax. Histograms shown in A and D were pooled data from two independent experiments with triplicates. Data were expressed as mean ± SEM and analyzed for statistical significance by *t* test (C) or Two-way ANOVA (A, D) (*$P < 0.05$; **$P < 0.01$; ***$P < 0.001$).

Table 1. AS1842856 abolishes AGK knockdown-induced sensitization effect to venetoclax. The molecular status of 33 DLBCL patients including the expression levels of BCL-2 determined by immunohistochemistry and BCL-2 translocation determined by FISH. + represents positive BCL-2 translocation; - represents negative BCL-2 translocation; ND represents not determined.

Figure S1

A

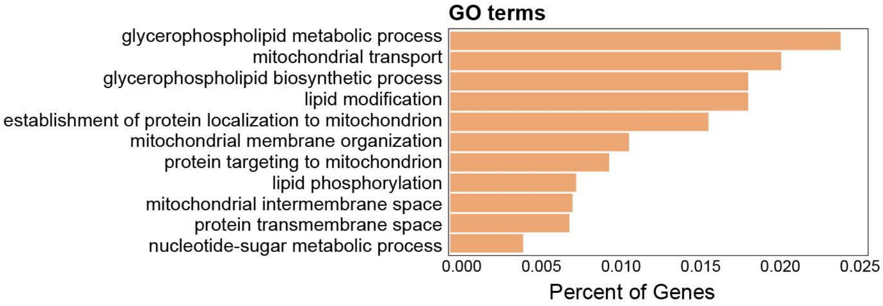
GO terms
glycerophospholipid metabolic process
mitochondrial transport
glycerophospholipid biosynthetic process
lipid modification
establishment of protein localization to mitochondrion
mitochondrial membrane organization
protein targeting to mitochondrion
lipid phosphorylation
mitochondrial intermembrane space
protein transmembrane space
nucleotide-sugar metabolic process
0.000
0.005
0.010
0.015
0.020
0.025
Percent of Genes

B

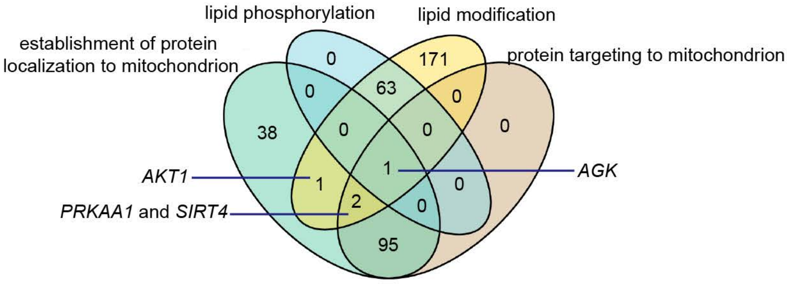
lipid phosphorylation
lipid modification
establishment of protein localization to mitochondrion
protein targeting to mitochondrion
0
171
0
63
0
38
0
0
0
AKT1
1
1
AGK
PRKAA1 and SIRT4
2
0
0
95

Figure S2

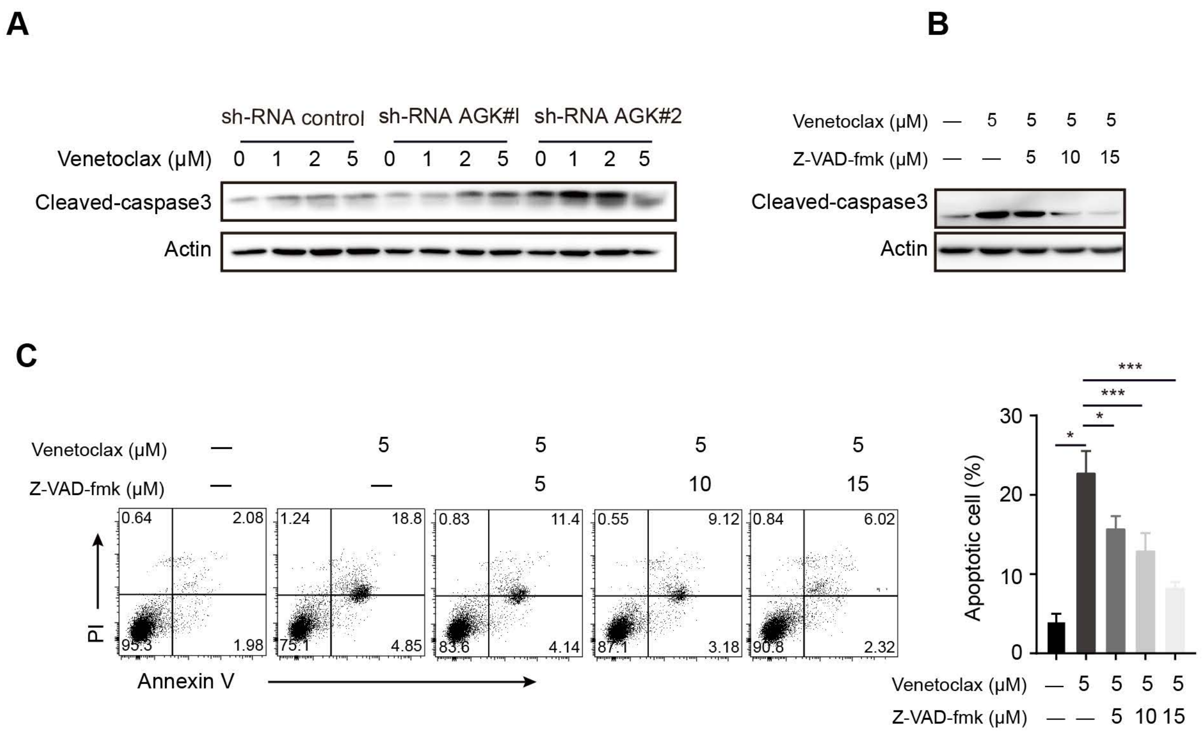
A
sh-RNA control
sh-RNA AGK#l
sh-RNA AGK#2
Venetoclax (μM) 0 1 2 5 0 1 2 5 0 1 2 5
Cleaved-caspase3
Actin
B
Venetoclax (μM) — 5 5 5 5
Z-VAD-fmk (μM) — — 5 10 15
Cleaved-caspase3
Actin
C
Venetoclax (μM) — 5 5 5 5
Z-VAD-fmk (μM) — — 5 10 15
0.64 2.08 95.3 1.98
1.24 18.8 75.1 4.85
0.83 11.4 83.6 4.14
0.55 9.12 87.1 3.18
0.84 6.02 90.8 2.32
PI
Annexin V
Apoptotic cell (%)
0
10
20
30
*
*
***
***
Venetoclax (μM) — 5 5 5 5
Z-VAD-fmk (μM) — — 5 10 15

Figure S3

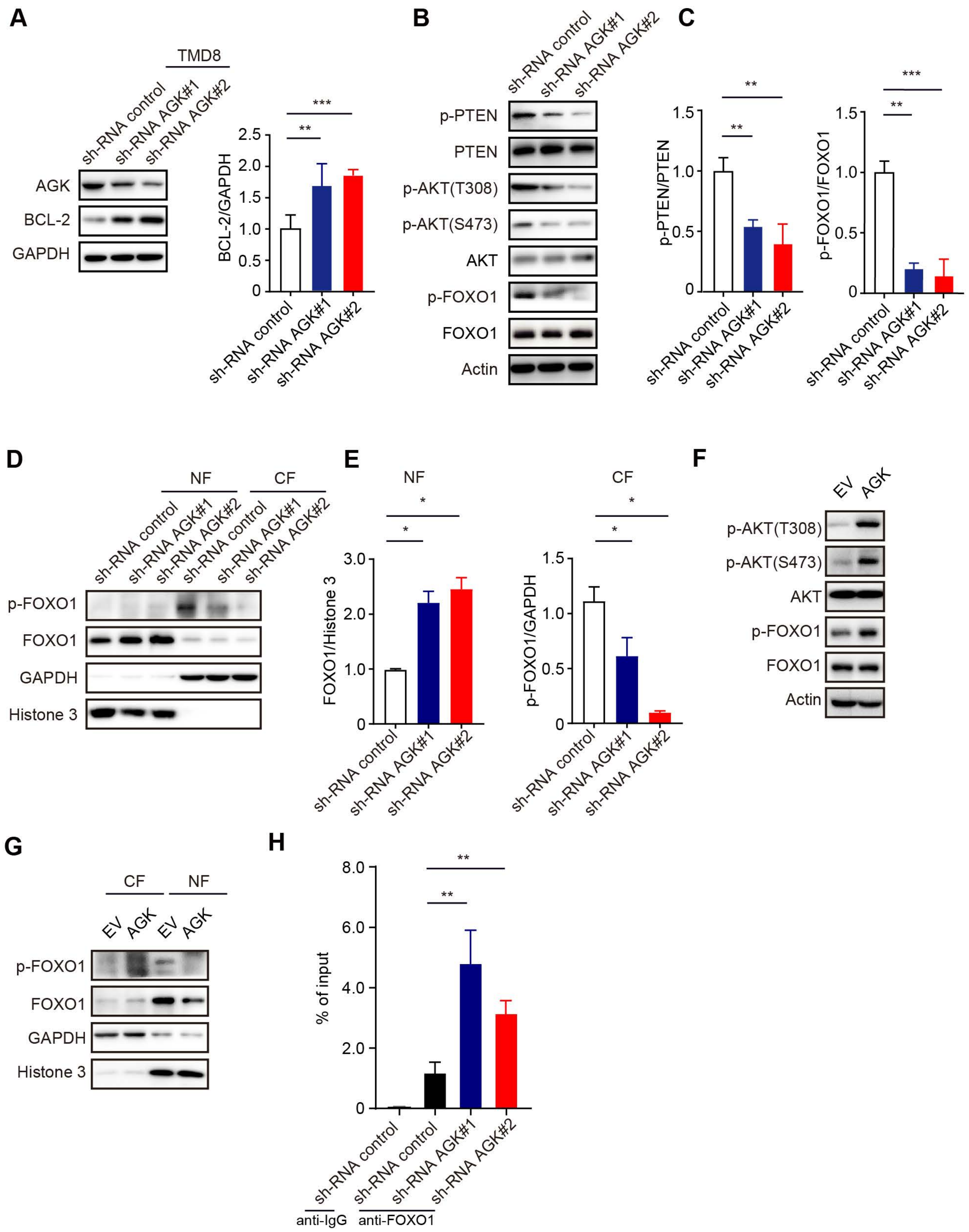

A
TMD8
sh-RNA control
sh-RNA AGK#1
sh-RNA AGK#2
AGK
BCL-2
GAPDH
BCL-2/GAPDH
B
p-PTEN
PTEN
p-AKT(T308)
p-AKT(S473)
AKT
p-FOXO1
FOXO1
Actin
C
p-PTEN/PTEN
p-FOXO1/FOXO1
D
NF
CF
Histone 3
E
FOXO1/Histone 3
p-FOXO1/GAPDH
F
EV
AGK
G
H
% of input
anti-IgG
anti-FOXO1

Figure S4

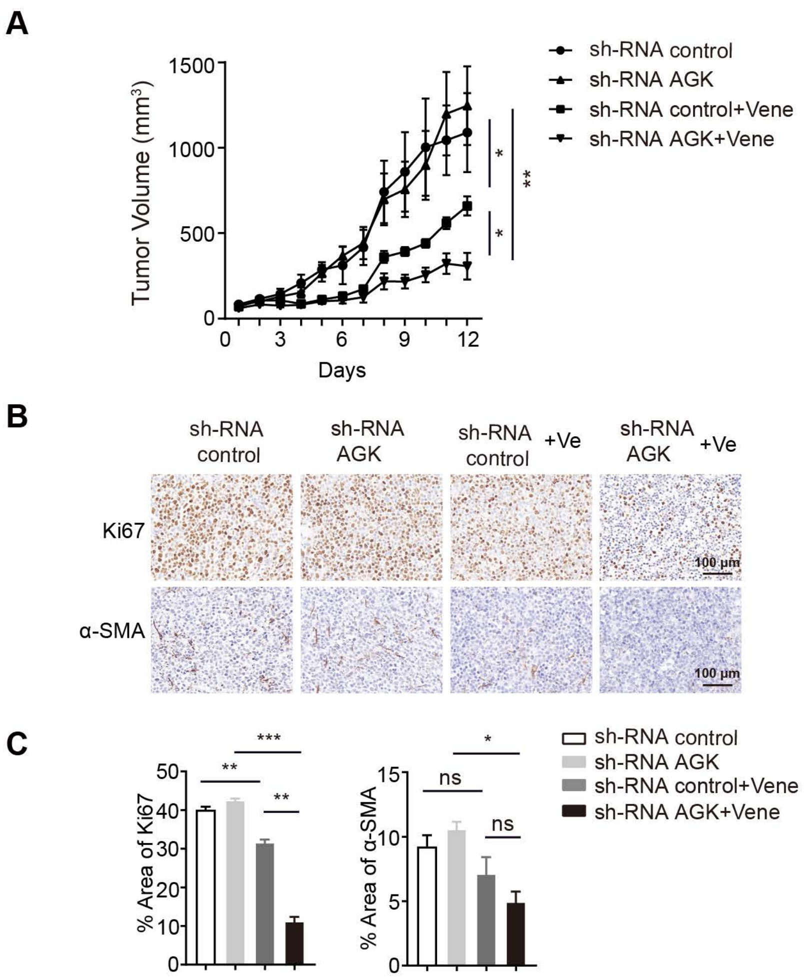
A
Tumor Volume (mm³)
1500
1000
500
0
0
3
6
9
12
Days
sh-RNA control
sh-RNA AGK
sh-RNA control+Vene
sh-RNA AGK+Vene
*
*
**
B
sh-RNA control
sh-RNA AGK
sh-RNA control +Ve
sh-RNA AGK +Ve
Ki67
α-SMA
100 μm
100 μm
C
% Area of Ki67
50
40
30
20
10
0
**
***
**
% Area of α-SMA
15
10
5
0
ns
*
ns
sh-RNA control
sh-RNA AGK
sh-RNA control+Vene
sh-RNA AGK+Vene

Figure S5

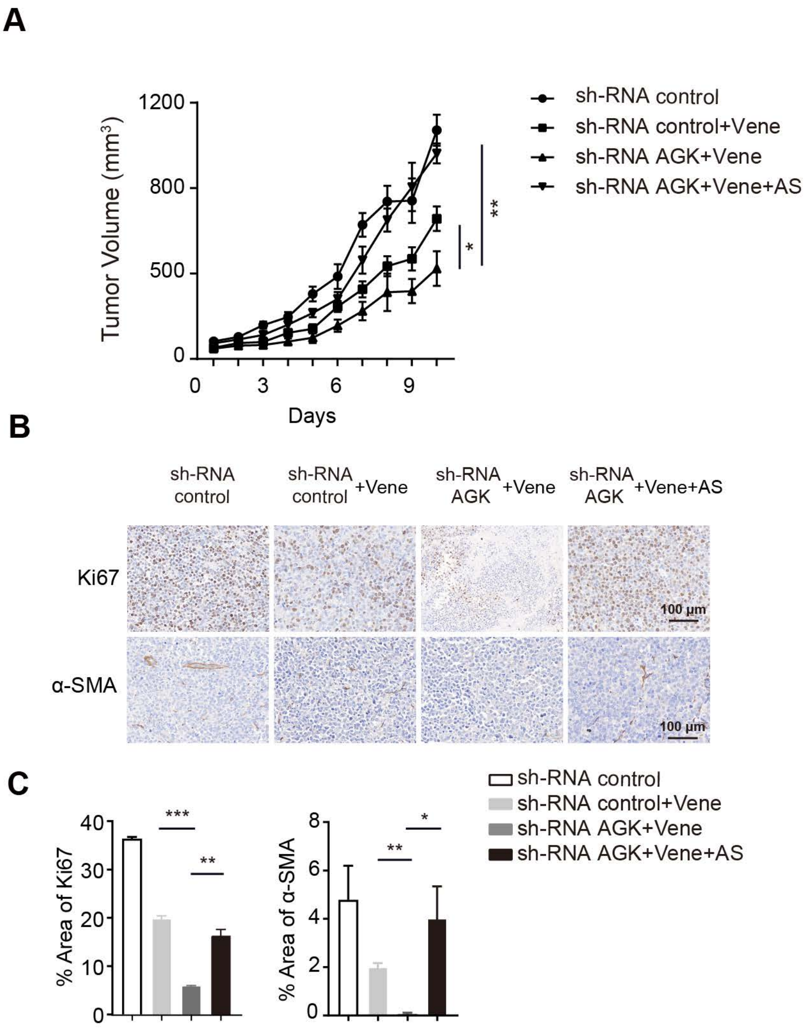
A
1200
800
500
0
0
3
6
9
Days
Tumor Volume (mm3)
*
**
sh-RNA control
sh-RNA control+Vene
sh-RNA AGK+Vene
sh-RNA AGK+Vene+AS
B
sh-RNA control
sh-RNA control +Vene
sh-RNA AGK +Vene
sh-RNA AGK +Vene+AS
Ki67
α-SMA
100 μm
100 μm
C
40
30
20
10
0
% Area of Ki67
***
**
8
6
4
2
0
% Area of α-SMA
**
*
sh-RNA control
sh-RNA control+Vene
sh-RNA AGK+Vene
sh-RNA AGK+Vene+AS

Figure S6

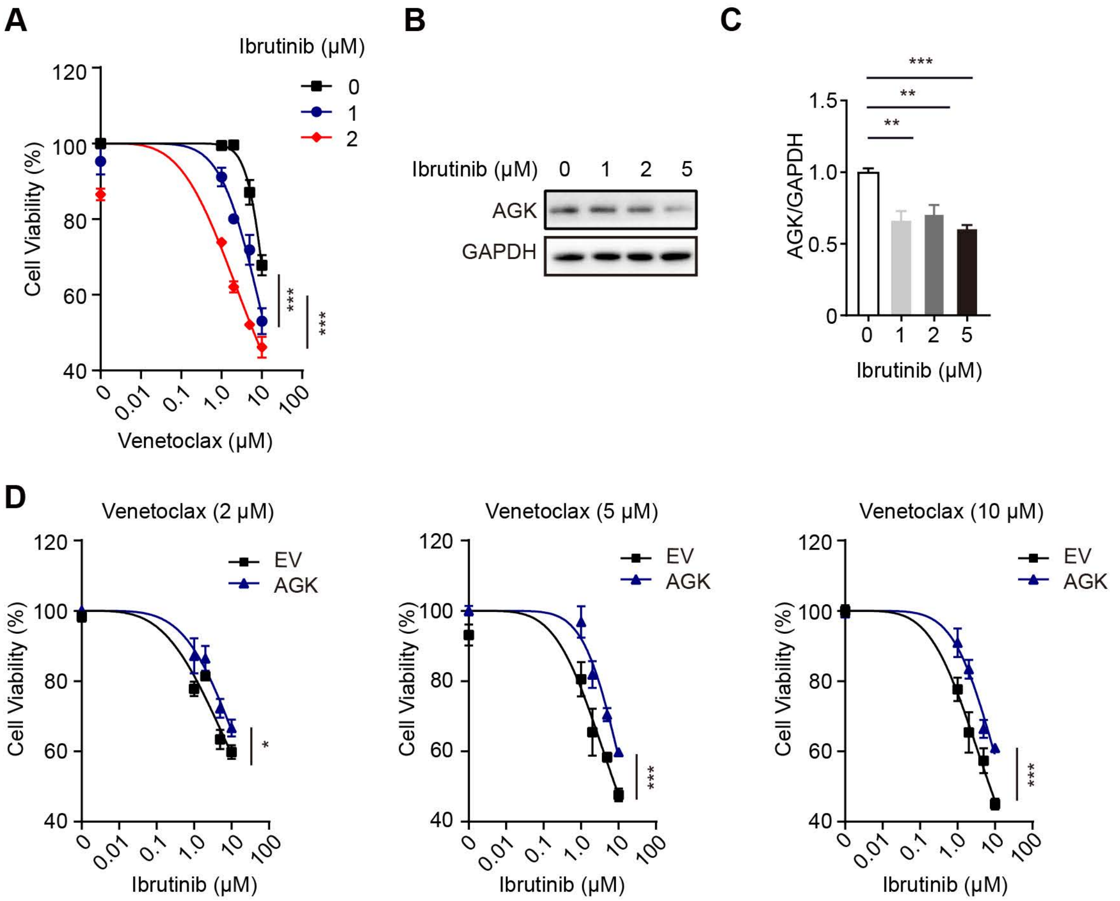
A
Ibrutinib (μM)
0
1
2
Cell Viability (%)
120
100
80
60
40
***
***
0
0.01
0.1
1.0
10
100
Venetoclax (μM)
B
Ibrutinib (μM) 0 1 2 5
AGK
GAPDH
C
AGK/GAPDH
1.5
1.0
0.5
0
***
**
**
0 1 2 5
Ibrutinib (μM)
D
Venetoclax (2 μM)
EV
AGK
*
Ibrutinib (μM)
Venetoclax (5 μM)
***
Venetoclax (10 μM)
***
